# Nilotinib-induced alterations in endothelial cell function recapitulate clinical vascular phenotypes independent of ABL1

**DOI:** 10.1038/s41598-024-57686-8

**Published:** 2024-03-26

**Authors:** Emily A. Pinheiro, Jean-Marc DeKeyser, Brian Lenny, Yadav Sapkota, Paul W. Burridge

**Affiliations:** 1 Department of Pharmacology, Northwestern University Feinberg School of Medicine, Chicago, IL 60611 USA; 2 Center for Pharmacogenomics, Northwestern University Feinberg School of Medicine, 320 E Superior St, Searle 8-525, Chicago, IL 60611 USA; 3 Department of Epidemiology and Cancer Control, St. Jude Children’s Hospital, Memphis, TN 38105 USA

**Keywords:** Induced pluripotent stem cells, Vascular diseases

## Abstract

Nilotinib is a highly effective treatment for chronic myeloid leukemia but has been consistently associated with the development of nilotinib-induced arterial disease (NAD) in a subset of patients. To date, which cell types mediate this effect and whether NAD results from on-target mechanisms is unknown. We utilized human induced pluripotent stem cells (hiPSCs) to generate endothelial cells and vascular smooth muscle cells for in vitro study of NAD. We found that nilotinib adversely affects endothelial proliferation and migration, in addition to increasing intracellular nitric oxide. Nilotinib did not alter endothelial barrier function or lipid uptake. No effect of nilotinib was observed in vascular smooth muscle cells, suggesting that NAD is primarily mediated through endothelial cells. To evaluate whether NAD results from enhanced inhibition of ABL1, we generated multiple ABL1 knockout lines. The effects of nilotinib remained unchanged in the absence of ABL1, suggesting that NAD results from off- rather than on-target signaling. The model established in the present study can be applied to future mechanistic and patient-specific pharmacogenomic studies.

## Introduction

Chronic Myeloid Leukemia (CML) is a myeloproliferative neoplasm caused by a reciprocal translocation between the Abelson murine leukemia (*ABL1*) gene on chromosome 9 and the breakpoint cluster region (BCR) on chromosome 22^1^. This results in production of the BCR-ABL1 fusion protein, which is constitutively active and causes unregulated production of abnormal granulocytes^1^. ABL1 tyrosine kinase inhibitors (TKIs) are a targeted class of drugs designed specifically to treat CML by inhibiting the kinase domain of ABL1 in the BCR-ABL1 fusion protein. Nilotinib was granted approval as a first line treatment for CML based on positive findings in a head-to-head clinical trial with the established first-line therapy imatinib. The 5- and 10-year data from this trial demonstrated nilotinib’s superiority to imatinib as a first-line treatment, as nilotinib induces a more rapid and substantial molecular response compared to imatinib^2–4^. Additionally, nilotinib was associated with a lower risk of progression to acute phase or blast crisis and lower rates of treatment-emergent BCR-ABL mutations^3^. Though nilotinib is highly effective, it is associated with significantly higher rates of cardiovascular events. These events are characterized by atherosclerosis across multiple arterial beds and include peripheral artery disease, ischemic heart disease, and ischemic cerebrovascular events^3^. In a head-to-head clinical trial, 23.5% of patients taking nilotinib developed a cardiovascular event compared to 3.6% of patients on an equivalent dose of imatinib^4^.

Why nilotinib causes arterial disease while imatinib, with its shared mechanism of action, does not is unknown. Nilotinib is a 10- to 50-fold more potent inhibitor of BCR-ABL1 due to enhanced inhibition of ABL1^5^. Previous studies have demonstrated that ABL1 inhibition reduces endothelial cell proliferation, which suggests a potential on-target mechanism for nilotinib’s adverse vascular effects^6^. However, nilotinib also differentially inhibits multiple off-target kinases that play key roles in vascular function, including TEK, SRC, KDR, FLT4, MAPK14, JAK1, and DDR1^7–9^. Which cell types are central to this adverse effect is similarly unknown. Atherosclerosis involves a complex interplay between two major vascular cell types—endothelial (ECs) and vascular smooth muscle cells (VSMCs)—in addition to contributions from the immune system^10^. Previous in vitro studies suggest a potential role for ECs in nilotinib-induced arterial disease (NAD) but did not examine the effects of nilotinib on VSMCs^8,11^ Mouse models of NAD have shown limited clinical translatability, with one study finding no effect of nilotinib on plaque size and another demonstrating an effect of nilotinib in a pairwise comparison but no significant difference overall across control, nilotinib, and imatinib^8,12^.

Human induced pluripotent stem cells (hiPSCs) are a powerful platform to study drug-induced adverse events, as they are a renewable source of cells that can be differentiated into multiple cell types of interest. hiPSCs recapitulate patient-specific adverse drug reactions and have been used to elucidate the mechanism of doxorubicin-induced cardiotoxicity in addition to allowing for validation of pharmacogenomic contributors to adverse event risk^13–15^. In the context of atherosclerosis, hiPSC-derived VSMCs have been used to identify factors that are atheroprotective for patients with type 2 diabetes mellitus^16^. Similarly, hiPSC-derived ECs have been used to understand the pathways activated by cigarette smoke that initiate atherogenesis^17^.

To date, the potential of hiPSCs to model NAD has not been studied. In this work, we harness the power of the hiPSC model to better understand the relative contributions of endothelial and vascular smooth muscle cells to NAD. We find that the effects of nilotinib are specific to endothelial cells and include decreased proliferation, decreased migration, and increased intracellular nitric oxide. Barrier function and lipid uptake are not affected by nilotinib. We next utilize this model to evaluate the mechanism of NAD. Using an ABL1 knockout (KO) approach, we demonstrate that differential ABL1 inhibition does not explain NAD and conclude that NAD results from off-target nilotinib-specific signaling. This model advances our understanding of the mechanism of NAD and provides a high-throughput platform for future studies of NAD pharmacogenomics.

## Results

### High-efficiency human endothelial cell differentiation and purification

**Figure 1:**
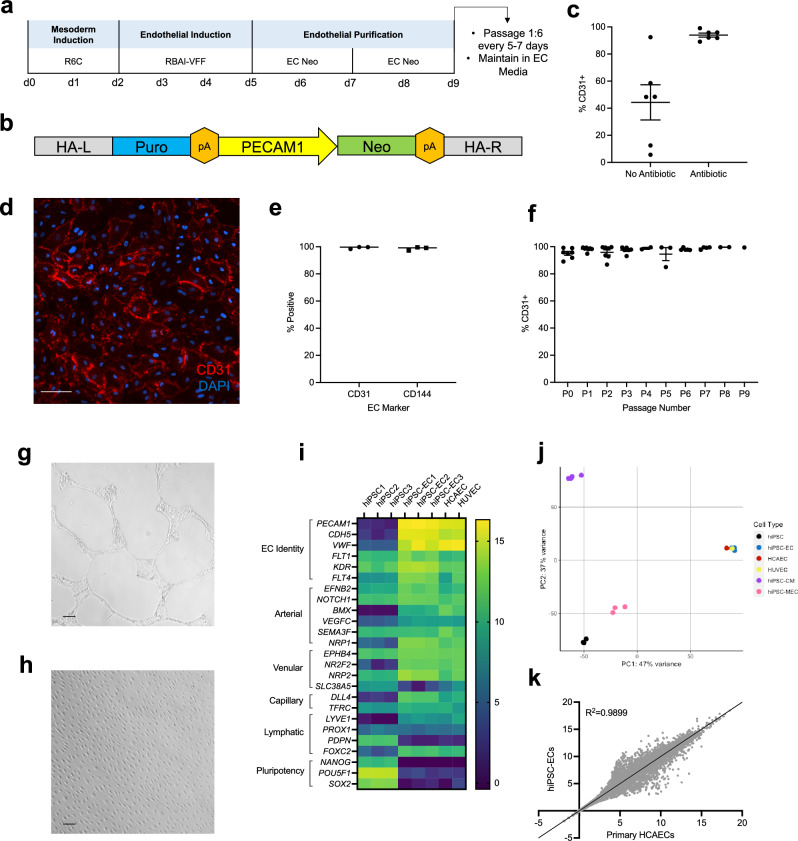
hiPSC-Derived Endothelial Cells Share Key Characteristics with Primary Cells. (**a**) Schematic of hiPSC endothelial cell differentiation (R6C—6 µM CHIR-99021 in RPMI; RBAI-VFF–RPMI with 2 mg/mL BSA, 200 µg/mL AA2P, 5 µg/mL insulin, 100 ng/mL VEGF-A, 100 ng/mL FGF2, and 10 µM forskolin; EC-Neo—in-house EC media with 100 µg/mL geneticin), (**b**) Schematic of donor vector for antibiotic selection of PECAM1-expressing cells (HA-L = left homology arm AAVS1, HA-R = right homology arm AAVS1, pA = polyadenylation site), (**c**) Flow cytometry assessment of EC purity in paired hiPSC-EC samples with and without neomycin purification (n = 6) Flow cytometry of hiPSC-ECs for EC surface markers (n = 3) (**d**) Immunofluorescent staining of hiPSC-ECs for CD31, (**e**) Flow cytometry of hiPSC-ECs for EC surface markers, (**f**) Flow cytometry assessment of EC purity across multiple passages after 4 days of neomycin selection at p0, (**g**) hiPSC-EC tube formation, (**h**) hiPSC-ECs align in response to shear stress after 48 h at 130 rpm on an orbital shaker, (**i**) Heatmap of RNASeq data for expression of EC subtype and pluripotency genes in hiPSCs, hiPSC-ECs, HUVECs, and HCAECs, (**j**) PCA plot of the top 500 most variable genes for hiPSC-ECs, HCAECs, HUVECs, hiPSCs, hiPSC-CMs, and hiPSC-MECs, (**k**) Correlation of RNASeq gene expression data between primary HCAECs and hiPSC-ECs. Scale bars represent 100 µM.

Human induced pluripotent stem cells (hiPSCs) offer a renewable source of cells for high-throughput disease and drug response modeling that is amenable to patient-specific genomic and pharmacogenomic studies. As EC dysfunction is a key characteristic of atherogenesis we established a protocol to differentiate hiPSCs to ECs (hiPSC-EC) (Fig. 1A) in order to study NAD in vitro. Our final differentiation protocol was established through optimization of hiPSC confluency at time of differentiation (Fig. S1A), mesoderm induction with CHIR-99021 (Fig. S1B,C), and endothelial cell induction and timing with VEGF, FGF2, and forskolin (Figure S1D–H). Following differentiation, we identified a matrix (Synthemax vitronectin) and medium (Supplemented MCDB/F12) that supports hiPSC-EC growth (Fig. S1I–K). To facilitate consistent, high-throughput hiPSC-EC purification, we designed and implemented an antibiotic-resistant *PECAM1* selection cassette (Addgene plasmid #201054; Fig. 1B). We demonstrated that 4 days of antibiotic selection is sufficient to yield a homogenous population of cells with high (> 98%) expression of the EC marker CD31, regardless of batch-to-batch variability in differentiation efficiency (Fig. 1C). These cells express canonical EC markers such as CD31 and CD144 at high levels (> 98%) (Fig. 1D,E) and maintain EC identity across several passages after a one-time antibiotic selection at P0 (Fig. 1F). In addition to identity markers, differentiated hiPSC-ECs express well-documented in vitro EC phenotypes, including tube formation and alignment to flow (Fig. 1G,H). We further characterized our hiPSC-ECs in comparison to commonly used primary EC models (human umbilical vein endothelial cells (HUVECs) and human coronary artery endothelial cells (HCAECs)) with RNA sequencing. hiPSC-ECs are transcriptionally similar to primary ECs in expression of EC identity genes and cluster with primary cells in a principal components analysis (Fig. 1I,J). There is 98.99% correlation in gene expression between hiPSC-ECs and primary HCAECs (Fig. 1K).

### Nilotinib decreases hiPSC-EC migration and proliferation and increases intracellular nitric oxide

**Figure 2:**
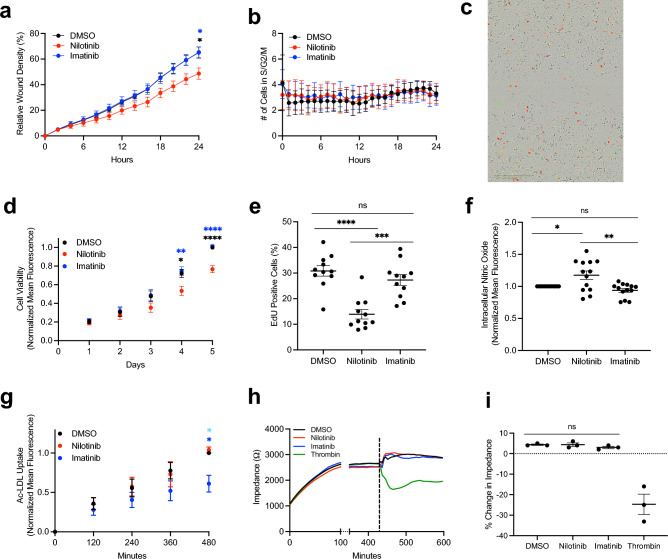
Nilotinib Impairs hiPSC-EC Migration, Proliferation, and Nitric Oxide Levels. (**A**) hiPSC-EC Wound Healing Assay in the presence of 3 µM Drug (n = 6), (**b** FUCCI Cell Cycle analysis of the number of cells in G2/Mitosis during the 24 h period following scratch (n = 5), (**c**) Representative image of FUCCI-transfected hiPSC-ECs at 24 h in the Scratch Assay. Red cells are those expressing CDT1 (G1) and green cells are those expressing Geminin (S/G2/M). (**d**) Resazurin-based assessment of hiPSC-EC Proliferation in the presence of 3 µM Drug (n = 7) **e,** EdU Nucleoside Analog Incorporation Assay in the presence of 3 µM Drug after 24-h pre-incubation (n = 11), (**f**) Flow cytometry analysis of DAF-FM intracellular nitric oxide staining after 72 h of 3 µM drug exposure (n = 13), (**g**) Quantification of Ac-LDL uptake in hiPSC-ECs in the presence of 3 µM drug using flow cytometry (n = 3), (**h**) Representative trace of hiPSC-EC impedance pre- and post-drug exposure. Dotted line represents time of drug addition, (**I**) Quantification of change in impedance 30 min after drug exposure (n = 3). *N* = biological replicates, unpaired Student’s T-test or one way ANOVA, *P ≤ 0.05, **P ≤ 0.01, ***P ≤ 0.001, ****P ≤ 0.0001, ns = not significant. Where stars are presented without lines, blue stars represent comparisons between nilotinib and imatinib, black stars between nilotinib and DMSO, and light blue stars between imatinib and DMSO.

We selected a series of in vitro assays that reflect various facets of EC dysfunction in atherosclerosis and compared nilotinib and imatinib to identify features that recapitulate the clinical difference in the effect of these drugs on the vasculature. A 3 µM dose was selected for both drugs to prevent confounding due to toxicity (N: LD_50_ = 61.9 µM, I: LD_50_ = 29.8 µM) (Fig. S2). This dose is also in line with the clinical C_max_ of both drugs (N: C_max_ = 4.3 µM, I: C_max_ = 5.3 µM). Migration and proliferation are key facets of angiogenesis and overall EC health. Nilotinib significantly decreased migration in a wound-healing scratch assay (N: 48.7% ± 4.4, I: 65.0% ± 4.4) (Fig. 2A). Because wound healing may represent a combination of migration and proliferation, we utilized a genetically encoded FUCCI cell cycle analysis system to monitor the cell cycle during the 24-h period of the scratch assay. We observed a low number (< 5 cells/timepoint) of cells in the S/G2/M portion of the cell cycle and this number did not differ by treatment (Fig. 2B,C). This suggests that mitosis was not a meaningful contributor to wound healing in the first 24 h and the effect of nilotinib is a result of decreased cell migration. We assessed proliferation using a 5-day resazurin assay as well as an EdU nucleoside analog incorporation assay. Nilotinib treatment significantly decreased proliferation in both the resazurin (Day 5 Relative to DMSO N: 0.77 ± 0.04, I: 1.02 ± 0.02) and EdU incorporation (N: 13.9% ± 1.81, I: 27.3% ± 2.12) assays (Fig. 2D,E). Given that atherosclerosis also involves disruptions in nitric oxide and lipid homeostasis, we measured intracellular nitric oxide and acetylated low-density lipoprotein (Ac-LDL) uptake in treated cells. Nilotinib significantly increased intracellular nitric oxide compared to DMSO or imatinib (Relative to DMSO N: 1.18 ± 0.07, I: 0.94 ± 0.03) (Fig. 2F). Nilotinib did not significantly alter Ac-LDL uptake compared to DMSO, though imatinib significantly decreased Ac-LDL uptake (Relative to DMSO N: 1.04 ± 0.04, I: 0.61 ± 0.11) (Fig. 2G). This finding is consistent with previous studies in macrophages as well as clinical trial data suggesting that imatinib may confer some degree of atheroprotection^18,19^. Finally, we assessed barrier function, which is known to be disrupted early in atherogenesis. We confirmed the capacity of this assay to assess disruptions in barrier function using thrombin, a known disruptor of EC barrier integrity. Neither nilotinib nor imatinib decreased barrier function, as measured by impedance on a multielectrode array (Fig. 2H,I).

### Nilotinib does not differentially affect hiPSC-VSMCs

**Figure 3:**
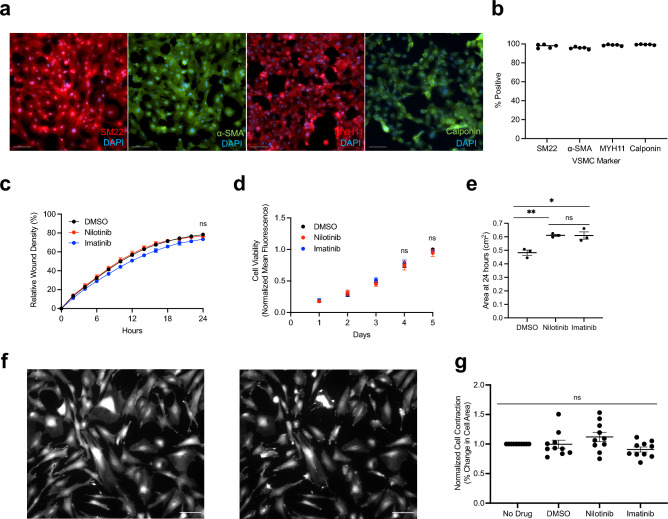
Nilotinib Does Not Differentially Affect hiPSC-VSMCs. **(a**) Immunofluorescence staining for vascular smooth muscle cell (SM22, α-SMA, MYH11, and Calponin) markers, (**b**) Flow cytometry analysis of VSMC markers **c**, hiPSC-VSMC Migration Assay in the presence of 3 µM Drug (n = 3), (**d**) Resazurin-based assessment of hiPSC-VSMC Proliferation in the presence of 3 µM Drug (n = 3), (**e**) Quantification of collagen gel area 24 h after plating (n = 3), (**f**) Representative images of Calcein-AM-stained cells pre- (left) and 20 min post 100 µM carbachol exposure (right), (**g**) Quantification of contraction after 72 h of 3 µM Drug using percentage reduction in cell area calculated by an ImageJ Macro (n = 10). *n* = biological replicates, unpaired Student’s T-test or one way ANOVA, *P ≤ 0.05, **P ≤ 0.01, ns = not significant. Scale bars represent 100 µM.

After identifying nilotinib-specific effects on ECs, we evaluated whether these findings extend to a second key vascular cell type, vascular smooth muscle cells (VSMCs). We adapted a published 4-day protocol to generate hiPSC-VSMCs.^16^ Our differentiated cells express high levels (> 95%) of VSMC markers including SM22, α-SMA, MYH11, and calponin (Fig. 3A,B). We selected assays to measure various facets of the contractile-to-synthetic phenotypic switch that occurs in atherosclerosis, which is characterized by increased proliferation and migration and decreased contraction^20^. Nilotinib did not impair VSMC migration or proliferation (Fig. 3C,D). In a collagen gel contraction assay both nilotinib and imatinib decreased VSMC contraction (Fig. 3E). Contraction was comparable between nilotinib- and imatinib-treated cells and thus is unlikely to explain the nilotinib-specific phenotype seen clinically. Neither nilotinib nor imatinib had any effect on VSMC contraction in response to carbachol (Fig. 3F,G).

### Differential ABL1 inhibition does not explain the nilotinib-specific EC phenotype

**Figure 4:**
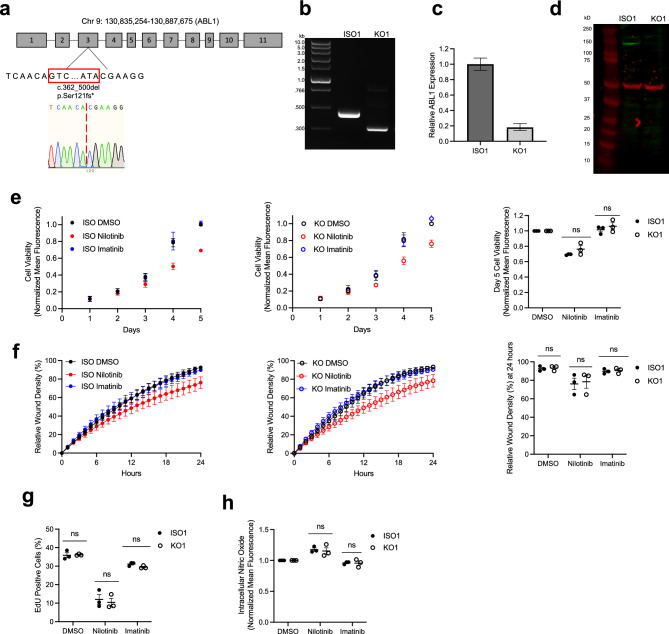
Characterization of the Effects of ABL1 Knockout in hiPSC-ECs. (**a**) Schematic and Sanger sequencing of ABL1-KO line (GRCh38), (**b**) DNA Gel Electrophoresis of Isogenic (ISO) and ABL1 KO cell lines, (**c**) RT-PCR of ABL1 expression normalized to ISO cell line, (**d**) Western blot for ABL1 in ISO and KO hiPSC-ECs, (**e**) Resazurin-based assessment of hiPSC-EC proliferation for ABL1 KO and ISO lines in the presence of 3 µM Drug (n = 3), (**f**) Comparison of Wound Healing in ABL1 KO and ISO hiPSC-ECs in the presence of 3 µM Drug (n = 3), (**g**) EdU Nucleoside Analog Incorporation Assay of ABL1 KO and ISO lines in the presence of 3 µM Drug after 24-h pre-incubation, (**h**) Flow cytometry analysis of DAF-FM intracellular nitric oxide staining in ABL1 KO and ISO hiPSC-ECs after 72 h of 3 µM drug exposure (n = 3). *n* = biological replicates, Unpaired Student’s T-test, ns = not significant.

The enhanced clinical efficacy of nilotinib in CML results from its more potent inhibition of ABL1 compared to imatinib^5,7^. We assessed whether this difference in ABL1 inhibition is responsible for the nilotinib-specific EC phenotype using an ABL1 KO model. We used a dual guide CRISPR/Cas9 approach to create a 139 bp frameshift deletion in *ABL1* exon 3 that was confirmed by Sanger sequencing and DNA gel electrophoresis (Figs. 4A,B, S4A). This genetic edit corresponded to decreased ABL1 RNA expression and absence of the ABL1 protein (Figs. 4C,D, S4B). We then ran isogenic and KO lines in parallel in all EC assays that had a nilotinib-specific difference. ABL1 KO did not impair proliferation, migration, or nitric oxide signaling (Fig. 4E–H). Additionally, the difference in effect between nilotinib and imatinib persisted in the absence of ABL1. These findings were replicated in two additional cell lines (Figs. S3, S4C,D).

## Discussion

The present study demonstrates that nilotinib directly affects multiple endothelial cell functions, including proliferation, migration, and nitric oxide homeostasis but not barrier function or Ac-LDL, and that these effects are independent of ABL1. EC capacity for migration and proliferation is necessary for maintenance of the vasculature, wound healing, and angiogenesis. Previous studies have demonstrated that reduced migration and proliferation in vitro correlate with endothelial etiologies of atherosclerosis, such as cigarette smoking and elevated glucose^21,22^. Thus, the decreased proliferation and migration observed with nilotinib exposure in our study are consistent with an anti-angiogenic endothelial mechanism of NAD.

Less intuitive is our finding of increased intracellular nitric oxide in nilotinib-treated cells. Nitric oxide has numerous functions in the vasculature including vasodilation and vasoprotection^23^. Endothelial dysfunction often results in impaired function of endothelial nitric oxide synthase (eNOS; *NOS3*) and decreased nitric oxide production^23^. In our cells increased NO may represent a compensatory increase in eNOS, similar to that observed both in vitro and in vivo with other pro-atherogenic conditions such as diabetes^24,25^. Alternatively, this increase may be driven by inducible NOS (iNOS; *NOS2*), which is activated in response to inflammation.

This study is the first to examine the effect of nilotinib on vascular smooth muscle cells. VSMCs are prominent in the development and advancement of atherosclerosis^26^. Lineage-tracing studies have demonstrated that even cells previously identified as macrophages and foam cells in atherosclerotic plaques are of VSMC origin, demonstrating the plasticity and centrality of these cells in plaque formation^26^. Beyond the role of VSMCs in atherogenesis broadly, some etiologies of atherosclerosis can be specifically localized to effects on VSMCs^16^. We assessed multiple VSMC functional characteristics that are altered in atherosclerosis and found no nilotinib-specific effects on migration, proliferation, or contraction. Although proliferation and migration were significantly reduced in ECs, no effect was seen in VSMCs for the assays reported, underscoring that these effects are cell-type specific and localize to ECs rather than VSMCs. Of note, the proliferation rate of VSMCs is lower compared to ECs, so it is possible that a more subtle differential effect of nilotinib would be evident in longer term culture.

With an ABL1 KO approach, we demonstrate that absence of ABL1 does not alter the EC phenotype. There was no difference in function for untreated ECs with ABL1 KO nor did elimination of ABL1 alter the difference in effect between nilotinib and imatinib. Because enhanced inhibition of ABL1 does not explain the differential effect of nilotinib on endothelial cells, this suggests that nilotinib alters endothelial cell function through off-target mechanisms. That nilotinib-induced endothelial effects is independent of ABL1 bodes well for future development of ABL1 kinase inhibitors for the treatment of CML. The efficacy of TKIs for CML is directly dependent on their ability to inhibit the ABL1 kinase domain of the BCR-ABL1 fusion protein and inhibit unregulated cell proliferation. If the degree of ABL1 inhibition were directly associated with the risk of adverse arterial events, this side effect could not be mitigated without adversely affecting efficacy. With this knowledge it is possible to envision a next generation of TKIs with equivalent or superior efficacy to nilotinib that does not adversely affect the vasculature.

To realize this potential, it will be necessary to identify the specific off-target mechanism of NAD. Multiple candidates known to affect vascular function are inhibited either selectively or more potently by nilotinib compared to imatinib. Cell-free kinase inhibition studies and proteomic analyses have identified differential inhibition by nilotinib of multiple targets including TEK, SRC, KDR, FLT4, MAPK14, JAK1, DDR1, KIT, and PDGFRB^7–9,27^. Which target(s) are central to the mechanism of NAD remains to be assessed in detail. Hadzijusufovic et al.^8^ explored this question with a pharmacologic screening approach. They pharmacologically inhibited MAPK14, KDR, TEK, BRAF, and JAK1 at concentrations up to the IC_50_ for each compound. They found no effect of any individual drug on EC proliferation at the concentrations tested, while a mixture of all five inhibitors appeared to replicate the effect of nilotinib on EC expansion. While preliminary, they use this data to suggest that NAD may result from inhibition of multiple targets rather than being attributable to a single off-target kinase. Our work demonstrates that nilotinib has multiple distinct phenotypic effects on ECs, and these distinct effects could either represent independent off-target mechanisms or result from inhibition of a common pathway. Of note, while off-target inhibition likely contributes to NAD pathogenesis, the effect of nilotinib on off-target pathways may be therapeutic in other diseases such as Sturge-Weber Syndrome^27^.

Defining the etiology of NAD will ultimately aid in its prevention. Proposed approaches to manage drug-induced cardiovascular toxicity typically fall into three categories: pharmacogenomic and/or risk-based screening of patients to preemptively avoid administration in patients at high risk, concomitant administration of a protective adjuvant therapy, and optimization of the compound structure to minimize adverse effects. To date, no pharmacogenomic studies have been conducted to identify which patients are disproportionately at risk for NAD. With respect to concomitant treatment, administration of proangiogenic therapy to patients with CML is clinically unfeasible. Patients with CML have an elevated risk of secondary malignancies compared to the general population and administration of proangiogenic therapy could potentially accelerate these malignancies^28^. Therefore, the most promising path forward in CML treatment will require identification of the specific off-target mechanism(s) of NAD and utilization of this information to design the next generation of ABL1 TKIs.

In parallel, the model developed in the present study can be used to identify pharmacogenomic contributors to NAD risk. Available clinical data demonstrates that patients are differentially susceptible to NAD and that this difference is not fully attributable to pre-existing cardiovascular risk factors^19,29^. This potentially suggests a genomic contributor to NAD risk, but to date no published studies have evaluated NAD pharmacogenomics. hiPSC models have been used both to functionally validate candidate genes identified in candidate gene or genome-wide association studies as well as to discover novel variants of interest^15,30^. Patient-specific hiPSC lines could be generated from individuals treated with nilotinib who did and did not develop NAD. Observation of patient-specific differences in hiPSC response to nilotinib would support a pharmacogenomic hypothesis of NAD. These cell lines could then be used in conjunction with whole genome sequencing to identify and validate variants of interest.

While our hiPSC-ECs are functionally and transcriptionally comparable to primary arterial and venous cells, there are limitations inherent in a two-dimensional static EC culture. This is evident, in part, from the transcription-level data demonstrating loss of distinct markers of arterial and venous identity even in primary cells. ECs are highly specialized, mechanosensitive cells that respond both to local forces such as shear stress and the organ-specific microenvironment^31^. These nuances are lost in EC-only, static culture systems. NAD occurs in multiple, distinct vascular beds (e.g., cerebral, renal, etc.), suggesting that organ-specific identity may be less crucial for this adverse effect. Whether nilotinib mediates known relationships between mechanical factors, such as laminar versus turbulent flow, and atherosclerosis, would require study in a dynamic system. Additionally, arterial disease is a complex process involving interactions between numerous cell types, which represents a limitation of single cell-type culture systems. Future studies could address these interactions with co-culture models in conjunction with pathological correlation with PAD-affected vessels. Despite these limitations, nilotinib-specific differences that recapitulate clinical differences between nilotinib and imatinib are still detectable with a static, EC-only approach. This system, too, has the advantage of being amenable to high-throughput screening.

In summary, we have demonstrated that hiPSC-derived vascular cells are an informative model for the study of nilotinib-induced vascular toxicity. We find that endothelial cells are a key site for nilotinib-induced cellular dysfunction, while no nilotinib-specific effect on vascular smooth muscle cells was detected. These effects are not mediated by ABL1 inhibition, which bodes well for the development of novel TKIs for treatment of CML with reduced vascular toxicity. The tools and assays developed in the present study can be employed in future mechanistic and patient-specific pharmacogenomic studies to avert NAD and advance precision medicine for patients with CML.

## Methods

### Human induced pluripotent stem cell culture

hiPSC lines were generated from healthy controls (4 male and 4 female). Protocols were approved by the Northwestern University Institutional Review Board and written informed consent was obtained from all volunteers prior to blood draw. All protocols were performed in accordance with relevant guidelines and regulations, including the Declaration of Helsinki. hiPSC lines were derived from isolated peripheral blood mononuclear cells using the CytoTune-iPS 2.0 Sendai Reprogramming Kit (Invitrogen, A16518) reprogramming protocol as previously described^32^. Cells were routinely maintained in B8 medium^32^ on 1:800 diluted growth factor reduced Matrigel (Corning, 356234). B8 was supplemented with 2 μM Rho kinase inhibitor (thiazovivin) for the first 24 h after passage. Cells were passaged at a ratio of 1:15 every 3 days using 0.5 mM EDTA, achieving 80% confluence. Cell lines were used between passages 20 and 90. All pluripotent and reprogramming cell cultures were maintained at 37 ºC in Heracell VIOS 160i direct heat humidified incubators (Thermo Scientific) with 5% CO_2_ and 5% O_2_. All cultures were maintained with 2 mL medium per 9.6 cm^2^ of surface area or equivalent. All cultures were routinely tested for mycoplasma using a MycoAlert PLUS Kit (Lonza, LT07-318) and a Varioskan LUX (Thermo Scientific) plate reader.

### Endothelial cell differentiation

hiPSCs were differentiated upon reaching ~ 50% confluence. B8 medium was changed to R6C, consisting of RPMI 1640 supplemented with 6 μM of the glycogen synthase kinase 3-b inhibitor CHIR-99021, to induce mesoderm formation. To induce EC identity, medium was changed on Day 2 to RBAI-VFF consisting of RPMI supplemented with 2 mg/mL of bovine serum albumin, 200 µg/mL of L-ascorbic acid-2-phosphate (AA2P), 5 µg/mL of recombinant human insulin, 100 ng/mL of VEGFA_165_, 100 ng/mL FGF2-G3 (made in-house), and 10 μM forskolin. On day 5, endothelial cells were dissociated and analyzed.

### Endothelial cell purification and culture

Differentiated hiPSC-ECs were cultured from D6 onwards in our in-house EC Medium consisting of a 1:1 volume of MCDB131 (Corning, 15-100-CV) and Ham’s F-12 supplemented with 5 ng/mL FGF2, 50 µg/mL AA2P, 1 µg/mL hydrocortisone, 2% Cosmic Calf Serum (CCS; Hyclone, SH3008703HI), 10 mM GlutaMAX (Gibco, 35–050-061), 15 ng/mL IGF1, 5 ng/mL EGF, 5 ng/mL VEGFA_165_, and 0.75 U/mL heparin. On day 5 of hiPSC-EC differentiation, cells were treated with 100 µg/mL Geneticin (Neomycin) in EC Medium for 4 days in order to achieve a pure hiPSC-EC population. Cells were then passaged 1:6 onto 6-well plates coated with a 1:320 dilution of Synthemax (Corning, 3535) in EC media without antibiotic. Cells were passaged 1:6 every 5–7 days. hiPSC-ECs were maintained at 37 °C in 5% CO_2_ and atmospheric (~ 21%) O_2_.

### Smooth muscle differentiation

hiPSCs were grown to ~ 60% confluence and differentiated to vascular smooth muscle cells according to an adapted version of protocols previously described^16^. On Day 0 cells were moved to normoxic incubators and exposed to R6C as above for two days to induce mesoderm formation. VSMC induction was then achieved with 2 days of RBAI-PA media consisting of RPMI supplemented with 2 mg/mL of BSA, 200 µg/mL of AA2P, 5 µg/mL of recombinant human insulin, 12.5 µM PDGF-BB, and 12.5 µM Activin A^16^. Vascular smooth muscle cells were dissociated on differentiation day 4 in Accutase for 6 min at 37 °C, centrifuged at 300 × *g* for 5 min, and analyzed.

### Smooth muscle cell culture

Differentiated hiPSC-VSMCs were passaged 1:6 onto Synthemax-coated 6-well plates. Cells were maintained in Human Vascular Smooth Muscle Basal Medium (Gibco, M231500) supplemented with Smooth Muscle Growth Supplement (Gibco, S00725). Cells were passaged 1:6 every 5–7 days. hiPSC-VSMCs were maintained at 37 °C in 5% CO_2_ and atmospheric (~ 21%) O_2_.

### Flow cytometry

For analysis of cell surface molecules live cells were incubated with antibody on ice for 30 min and washed prior to data collection. For intracellular staining, cells were fixed with 4% paraformaldehyde (PFA) in DPBS (100 µL) for 20 min. Cells were then washed twice using DPBS and permeabilized in 100 µL of DPBS with 0.5% BSA and 0.5% saponin for 15 min. Once fixed and permeabilized, cells were stained for 2 h at room temperature and washed prior to data collection. When unconjugated antibodies were used, cells were stained with the primary antibody for 2 h at room temperature, washed, and then stained with the secondary antibody for 1 h. All data were collected using a CytoFLEX flow cytometer (Beckman Coulter) and analyzed using CytExpert 2.2 software (Beckman Coulter) using isotype controls to set positive gates. Antibodies are listed in Supplemental Table 1.

### Quantitative real-time PCR

Cell lysates for RT-qPCR were collected in 350 μL TRIzol (Invitrogen, 15596026) and total RNA was isolated using Direct-zol RNA Microprep kit (Zymo Research, R2062), following the manufacturer’s protocol. Reverse transcription was performed from 1 μg of RNA using Maxima H Minus master mix (Thermo, M1662), following the manufacturer’s protocol. cDNA was then diluted 1:10. RT-qPCR was performed using Taqman gene expression assays (Applied Biosystems; GAPDH Hs02786624_g1 and ABL1 Hs01104728_m1) and Taqman Fast Advanced Master Mix (Applied Biosystems, 4444964) following the manufacturer’s protocol. All PCR reactions were prepared in triplicate in a 384-well format. Data were collected using the QuantStudio 5 Real-Time PCR system. Relative quantification of gene expression was calculated using the 2^−ΔΔCt^ method^33^, normalized to the reference GAPDH and wild-type control sample.

### Immunofluorescence

Cells were plated at 20,000 cells per well in Synthemax-coated 8-well microchamber slides. Cells were fixed with 4% PFA in DPBS for 15 min at RT, permeabilized with 1% saponin in DPBS for 15 min at RT, blocked with 3% BSA in DPBS for 30 min at RT, and stained with primary antibodies overnight at 4 °C (Supplemental Table 1). Cells were washed and then stained with secondary antibodies for 1 h at RT in the dark. Cells were washed three times. NucBlue (Invitrogen, R37606) was added during the last wash. Slides were imaged with a Ti-E inverted fluorescent microscope (Nikon Instruments) and a Zyla sCMOS camera (Andor) using NIS-Elements 4.4 Advanced software.

### Construction of the *PECAM1* plasmid

The pAAVS1-Puro-PECAM1-Neo plasmid (Addgene plasmid #201054) was made by digesting the mouse *PECAM1* promoter from PECAM1-eGFP^34^ using the AscI and NotI sites and ligating it into the backbone of an AAVS1-targeting plasmid derived from pAAVS1-P-CAG-DEST^35^ that had been modified by replacing the gateway destination cassette with a promoterless mScarlet open reading frame flanked by 5′ AscI and NotI sites and a 3′ SbfI site. A neomycin phosphotransferase open reading frame PCR-amplified with primers containing a 5′ NotI and 3′ SbfI site was cloned via TOPO cloning into the pCR4Blunt II-TOPO (Invitrogen, K280002) and used to replace the mScarlet reading frame using the NotI and SbfI sites. Ligations were transformed via heat shock to competent Stbl2 cells and clones were initially screened by restriction analysis using PvuII. Clones with the expected gel pattern were sequenced across the entirety of the *PECAM1* promoter and neomycin cassette via Sanger sequencing. To create the blasticidin resistant version of this plasmid the Puro resistance reading frame was removed and replaced with the Blasticidin ORF from a previously made AAVS1-Blast plasmid using PmII and SpeI restriction sites. PECAM1-eGFP was a gift from Victoria Bautch (Addgene plasmid # 14688; https://www.addgene.org/14688/; RRID:Addgene_14688). pAAVS1-P-CAG-DEST was a gift from Knut Woltjen (Addgene plasmid # 80490; https://www.addgene.org/80490/; RRID:Addgene_80490).

### Plasmid transfection

To introduce pAAVS1-Puro-PECAM1-Neo and pAAVS1-Blast-PECAM1-Neo cassettes into hiPSC lines, 5 × 10^6^ cells were electroporated with 1 µg of the KW516 plasmid ^35^ and 3 µg of the pAAVS1-Puro-PECAM1-Neo or pAAVS1-Blast-PECAM1-Neo plasmid. Cells were then selected in either 0.5 µg/mL puromycin or 2 µg/mL blasticidin for a minimum of 4 days. Selection was performed in parallel on cells transfected with the KW516 plasmid only, which had no antibiotic resistance, as a negative control.

### Cell migration analysis

25,000 cells/well were plated in a Synthemax-coated Incucyte Imagelock 96-well plate (Sartorius, BA-04856). 6 h after plating, when cells were fully adhered, a uniform scratch was introduced with the WoundMaker (Essen Biosciences). Cells were monitored for 24 h on the IncuCyte ZOOM system (Essen BioScience) for visualization and automated quantification.

### FUCCI cell cycle analysis

15,000 hiPSCs previously transfected with the pAAVS1-Blast-PECAM1-Neo cassette were plated in a 24-well Matrigel-coated plate. After 24 h cells were transduced with the Incucyte Cell Cycle Green/Red Lentivirus Reagent (Sartorius, 4779) at an MOI of 3 with 8 µg/mL Polybrene. After 24 h media was changed. Once confluent, cells were passaged to a 6-well plate and were cultured in B8 media with 0.5 µg/mL puromycin for a minimum of 4 days to select for stably transduced cells. Non-transduced cells were treated with puromycin as a negative control. Established FUCCI-transfected lines were then differentiated to endothelial cells using our established protocol and assayed on the IncuCyte ZOOM system (Essen BioScience) for visualization and automated quantification.

### Resazurin proliferation assay

hiPSC-ECs were seeded on 96-well Synthemax-coated plates at a density of 4000 cells/cm^2^. After cell adherence (~ 6 h), drug was added. Media changes occurred every two days. On each day of analysis, cells were assayed with resazurin. Resazurin sodium salt (Thermo, 418900050) was diluted to 5 mg/mL in sterile Milli-Q ultrapure water to generate a 10 × stock. Stock was diluted in FluoroBrite DMEM (Gibco, A1896702) with 0.3% BSA to 1x. 100 μL was added to each well of a 96-well plate and incubated at 37 °C for 3 h. Plates were read on a Varioskan Lux in fluorescent mode using top read and an excitation wavelength of 560 nm and an emission wavelength of 590 nm.

### EdU incorporation

20,000 cells/well were seeded on Synthemax-coated 6-well plates. After two days the media was changed to the treatment of interest for 24 h. Next, cells were incubated with 10 µM EdU for 2 h. Cells were collected, fixed, and stained per manufacturer’s instructions using the Click-iT EdU Alexa Fluor 647 Flow Cytometry Assay Kit (Invitrogen, C10419). Stained cell data was collected on a CytoFLEX flow cytometer (Beckman Coulter) and analyzed using CytExpert 2.2 software (Beckman Coulter) using a stained No EdU control to set positive gates.

### Barrier function

hiPSC-ECs were seeded on Synthemax-coated CardioExcyte96 0.6 mm Sensor Plates (Nanion, 201002) at a density of 25,000 cells/cm^2^. The plate was placed on the CardioExcyte 96 (Nanion) immediately after cell seeding. Impedance measurements were collected every 15 min. Seven h after plating, drug was added directly to the wells at a 2× concentration of equal volume for a final concentration of 1x. Measurements were collected every 5 min for the first hour following addition of drug and every 15 min thereafter. Impedance data was analyzed on DataControl96 software (V1.8.0).

### Tube formation assay

A 24-plate was coated with undiluted Matrigel at a volume of 200 µL/cm^2^. hiPSC-ECs were dissociated in Accutase for 6 min at 37 °C and seeded in Matrigel at a density of 40,000 cells/cm^2^. Cells were incubated at 37 °C for 16 h and imaged with a Ti-E inverted fluorescent microscope (Nikon Instruments) and a Zyla sCMOS camera (Andor) using NIS-Elements 4.4 Advanced software.

### Intracellular nitric oxide

25,000 cells/well were seeded in a Synthemax-coated 96-well plate. After 3 days of drug treatment media was changed to EC Medium with 1 µM DAF-FM Diacetate (4-Amino-5-Methylamino-2',7'-Difluorofluorescein Diacetate) (Invitrogen, D23844) and incubated at 37 °C for 20 min. Cells were washed with EC Medium and then incubated in fresh EC Medium at 37 °C for 15 min. Cells were analyzed on a CytoFLEX flow cytometer (Beckman Coulter) using CytExpert 2.2 software (Beckman Coulter).

### Ac-LDL uptake

25,000 cells/well were seeded in a Synthemax-coated 96-well plate. The following day media was replaced with serum-free EC Medium with 0.3% BSA for 24 h. Media was then changed to FluoroBrite DMEM with 0.3% BSA. At each timepoint, 5 µg/mL AlexaFluor 488 conjugated Ac-LDL from human plasma (Invitrogen, L23380) and the relevant concentration of the drug of interest was added. At the end of the experiment media was changed to cold FluoroBrite DMEM with 0.3% BSA. Cells were analyzed on a CytoFLEX flow cytometer (Beckman Coulter) and analyzed using CytExpert 2.2 software (Beckman Coulter).

### VSMC carbachol-induced contraction

2,000 cells/well were seeded in a Synthemax-coated 96-well plate and exposed to drug for 3 days. Cell media was then changed to Hanks Balanced Salt Solution (HBSS) with 2 µM Calcein-AM (Invitrogen, C1430). 6 images were obtained per well on the Vala Kinetic Imaging Cytometer. After imaging, an equal volume of warmed 200 µM Carbachol (Tocris, 2810) in HBSS was added directly to the wells for a final concentration of 100 µM. After 20 min, the same well areas were re-imaged. Images were analyzed in ImageJ using the “Triangle” auto-threshold algorithm.

### VSMC collagen gel contraction assay

The collagen gel contraction assay was conducted according to a published protocol^36^. A 24-well plate was pre-coated with CCS for 30 min before staining. A 4 mg/mL purified rat tail type I telo-collagen solution was combined 1:10 with the neutralization solution (Advanced Biomatrix, 51531KIT) and diluted to 1 mg/mL collagen with VSMC media containing the drug of interest and 1 ×  10^5^ hiPSC-VSMCs. CCS was removed from the wells and the collagen/cell suspension mixture was added to each well. The gel was placed at 37 °C for 20 min to allow for polymerization. A P200 pipette tip was gently passed around the edge of the gel to ensure detachment. 500 µL of media with the relevant drug condition or 100 µM Y-27632 (negative control) was added to each well. Wells were imaged 24 h later with an Olympus MVX10 microscope at 1.25 × magnification using a 0.63 × lens and dark field illumination. Images were captured with an Andor Neo sCMOS camera using Micro-Manager 2.0 software. Gel size was quantified in ImageJ.

### CRISPR/Cas9 genome editing

Guide RNAs were designed using a combination of Benchling and CRISPOR design tools. Custom guide-specific Alt-R CRISPR-Cas9 crRNA (IDT) and Alt-R CRISPR-Cas9 tracrRNA (IDT) were reconstituted in nuclease free duplex buffer to a concentration of 200 µM. cRNA and tracrRNA were combined 1:1 (final duplex concentration of 100 µM) and heated at 95 °C for 5 min. After allowing the duplex to cool to room temperature, it was combined with Alt-R S.p. HiFi Cas9 Nuclease V3 (IDT, 1081061) to achieve a molarity ratio of 2:1 Cas9:Duplex in the final volume. The Cas9 and RNA duplex were incubated at RT for 20 min to allow formation of the RNP complex. hiPSCs were dissociated in TrypLE for 3 min at RT. 1 × 10^6^ cells were electroporated with the RNP complex prepared above and Alt-R Electroporation Enhancer (IDT, 1075916) at a final concentration of 100 µM. To screen specific cRNA for efficacy, pooled cells were collected 4 days after transfection for DNA extraction and sequencing to confirm cutting. To establish KO lines, hiPSCs were transfected with two RNP complexes simultaneously, which contained validated cRNA to cut at two locations in the same exon. Transfected cells were seeded at a low density (divided among 12–24 wells of 6-well plates). 3–5 days after transfection clones were picked manually with a P20 pipette. Clones were sequenced at the following passage to identify candidate KOs. Guides and primers are listed in Supplemental Table 2.

### Western blot

Cells were seeded at 10,000 cells/cm^2^ on Synthemax-coated 10 cm^2^ tissue culture plates. Once confluent, cells were lysed in 200 µL Laemlli Sample Buffer. Samples were sonicated 5 times at 15 kHz for 5 s each. 45 µL of sample was combined with 5 µL of 400 mM, pH 8.0 Tris(2-carboxyethyl)phosphine hydrochloride. Samples were boiled on a hot plate at 100 °C for 2 min. Precision Plus Protein Dual Color Standards (Biorad 1,610,374) and the prepared samples were loaded onto a NuPAGE 4 to 12%, Bis–Tris, 1.0 mm, Mini Protein Gel (Invitrogen NP0321). Samples were run at 200 V for 30 min. Wet transfer to a PVDF membrane was performed at 30 V for 16 h at 4 °C overnight. The membrane was washed three times in TBST. Next, the membrane was blocked in 5% milk in TBST for 1 h at RT and then incubated at 4 °C overnight with primary antibodies (Supplemental Table 1). The membrane was then washed three times with TBST and incubated with secondary antibodies for 2 h at RT. The membrane was rinsed three times in TBST and imaged on an Odyssey CLx infrared imaging system with ImageStudio software.

### RNA–seq gene expression analysis

Passage 2 hiPSC–ECs from 3 individuals, HUVECs (Lonza, C2519A), and HCAECs (Lonza, CC-2585) were cultured to confluence in EC Medium, EGM-2 (Lonza, CC-3162), and EGM-2MV (Lonza, CC-3202), respectively. Primary cells were cultured per supplier instructions. Cell lysates were collected in 350 μL TRIzol and total RNA was isolated using Direct-zol RNA Microprep kit, following the manufacturer’s protocol including on-column DNase digestion to remove genomic DNA. Samples were quantified using an Agilent 2100 Bioanalyzer and passed QC. RNA Sequencing was performed by BGI on their DNBSEQ platform, generating ~ 40 million single-end 100 bp reads for each sample. Reads were mapped to the GRCh38 reference human genome using Subread software^37^. Gene expression levels were estimated using featureCounts function in the Subread software^38^. PCA plots were generated in R (v4.1.1).

### Statistical methods

Data were analyzed and graphed in GraphPad Prism 9. Detailed statistical information is included in the corresponding figure legends. Data were presented as mean ± SEM. Data was checked for normal distribution and comparisons were conducted via one way–ANOVA test or an unpaired two-tailed Student’s t-test with significant differences defined as *P* < 0.05 (*), *P* < 0.01 (**), *P* < 0.005 (***), and *P* < 0.0001 (****). No statistical methods were used to predetermine sample size.

### Study approval

This research was approved by the Northwestern University IRB. All subjects provided written informed consent prior to enrollment.

### Supplementary Information

Supplementary Information.

## Data Availability

RNA Sequencing files are available through the GEO repository (Accession #GSE229556).
