# Supplementary material for: Nilotinib-induced alterations in endothelial cell function recapitulate clinical vascular phenotypes independent of ABL1
Source: Sci Rep. 2024 Mar 26;14:7123. doi: 10.1038/s41598-024-57686-8 (PMC10966048; doi:10.1038/s41598-024-57686-8)
Supplement: Supplementary file 1 — Supplementary Information. [file 41598_2024_57686_MOESM1_ESM.pdf]

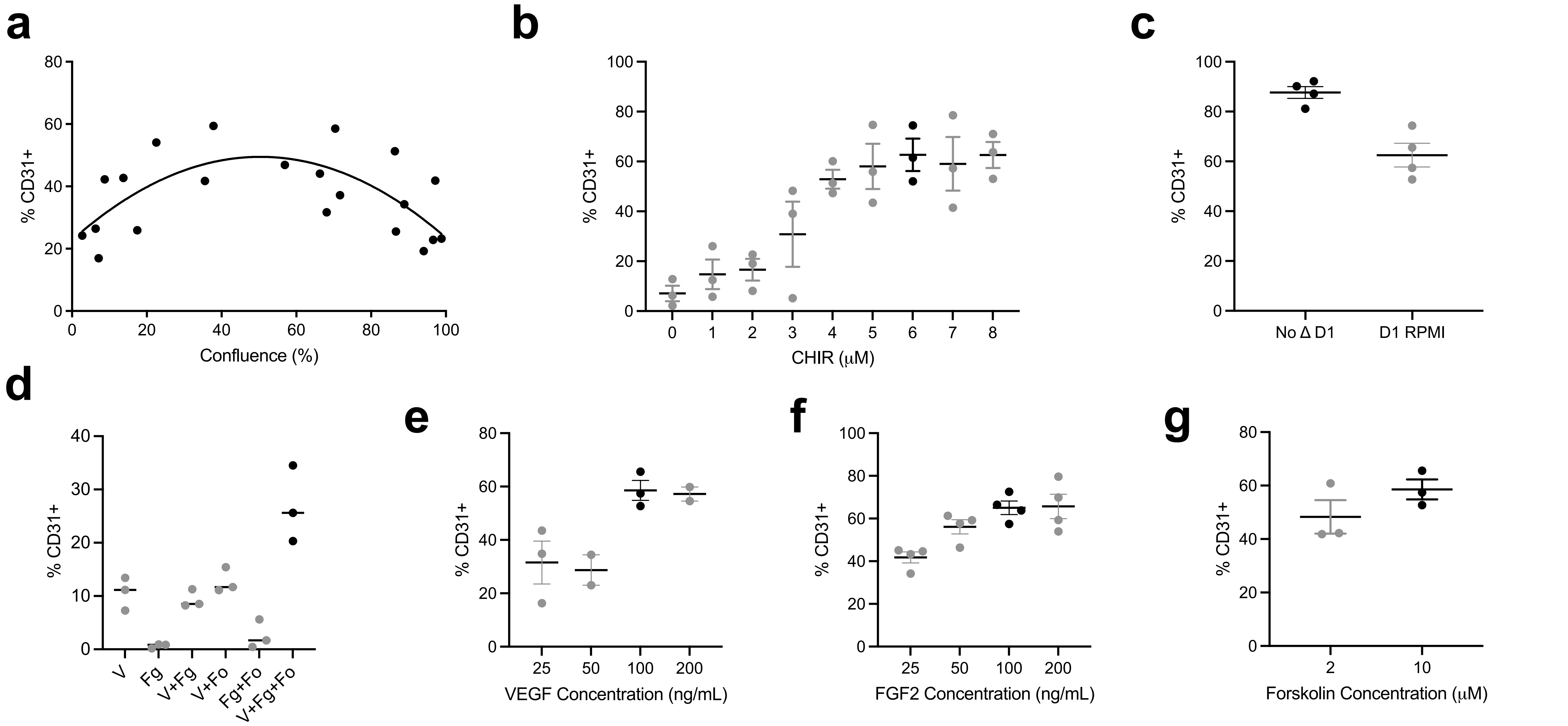

h

	D0	D1	D2	D3	D4	D5	D6
**1**	R6C		VFF	VFF		**Flow**	
**2**	R6C		VFF	VFF	**Flow**		
**3**	R6C		VFF		**Flow**		
**4**	R6C		VFF		VFF	**Flow**	
**5**	R6C		VFF	VFF	VFF	**Flow**	
**6**	R6C		VFF			**Flow**	
**7**	R6C		VFF	VFF	VFF		**Flow**
**8**	R6C		VFF		VFF		**Flow**

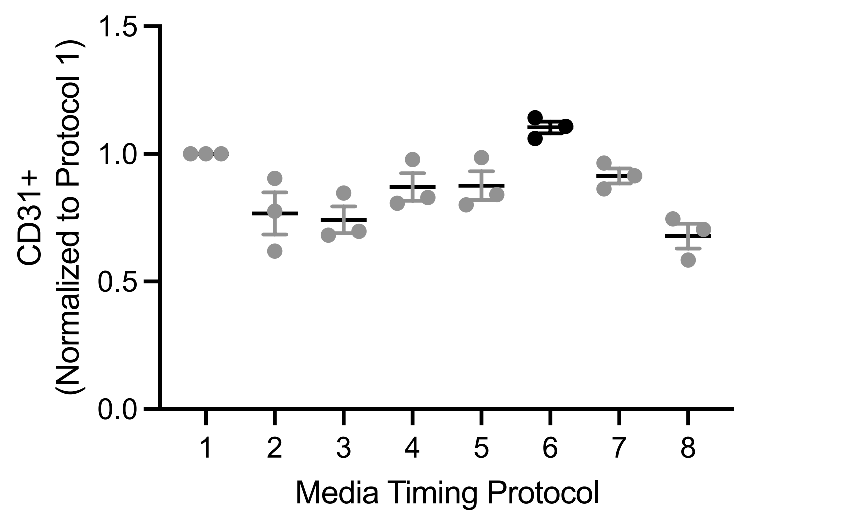

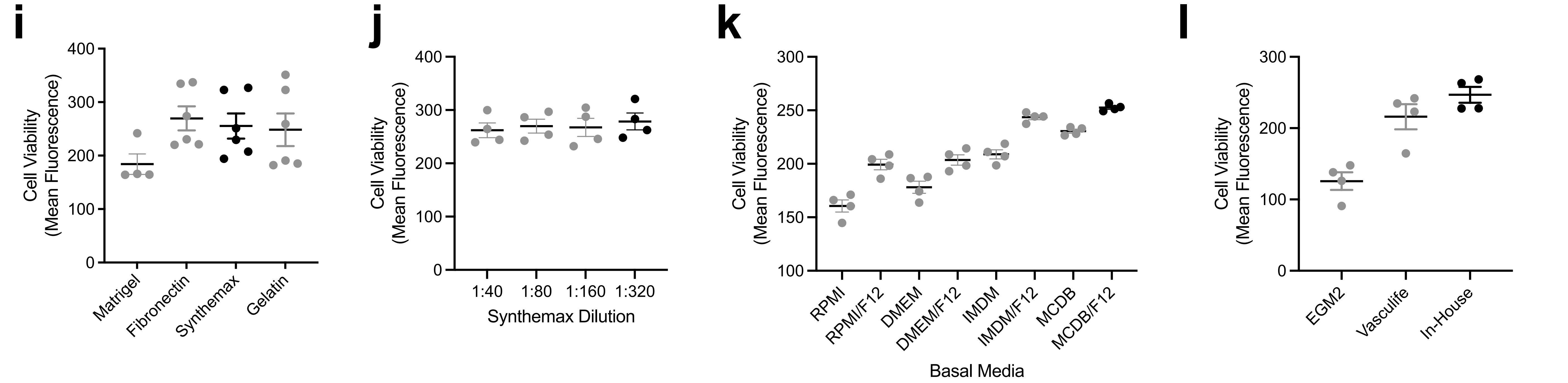

Supplementary Figure 1: Optimization of hiPSC-EC Differentiation and Growth. a, Flow cytometry assessment of the relationship between hiPSC confluency (as determined by an ImageJ algorithm) and EC purity across 7 split ratios (n=3) **b**, Flow cytometry of varying CHIR-99021 concentration for mesoderm induction at D0 of hiPSC-EC differentiation (n=3) **c,** Flow cytometry comparison of EC purity with no media change on D1 compared to a media change to RPMI (n=4) **d**, Flow cytometry assessment of EC purity with various combinations of VEGF 25 ng/mL (V), FGF2 25 ng/mL (Fg), and forskolin 2 μM (Fo) on D2 and D3 of differentiation (n=3) **e,** Flow cytometry assessment of EC purity with varying concentrations of VEGF (n=3), **f**, Flow cytometry assessment of EC purity with varying concentrations of FGF2 (n=4), **g**, Flow cytometry assessment of EC purity with varying concentrations of forskolin (n=3), **h**, Flow cytometry comparison of 8 different timing and duration permutations of RBAI-VFF media (each box represents a media change; n=3), **i**, Resazurin-based assessment of EC proliferation after 5 days of growth on various matrices (n=4), **j**, Resazurin-based assessment of EC proliferation after 5 days of growth on various concentrations of Synthemax (n=4), **k**, Resazurin-based assessment of EC proliferation after 5 days of growth in various basal media (where 2 media are listed the ratio is 1:1; n=4), **l**, Resazurin-based assessment of EC proliferation after 5 days of culture in our finalized in-house media formulation compared to two widely used commercial media (n=4). *n* = biological replicates

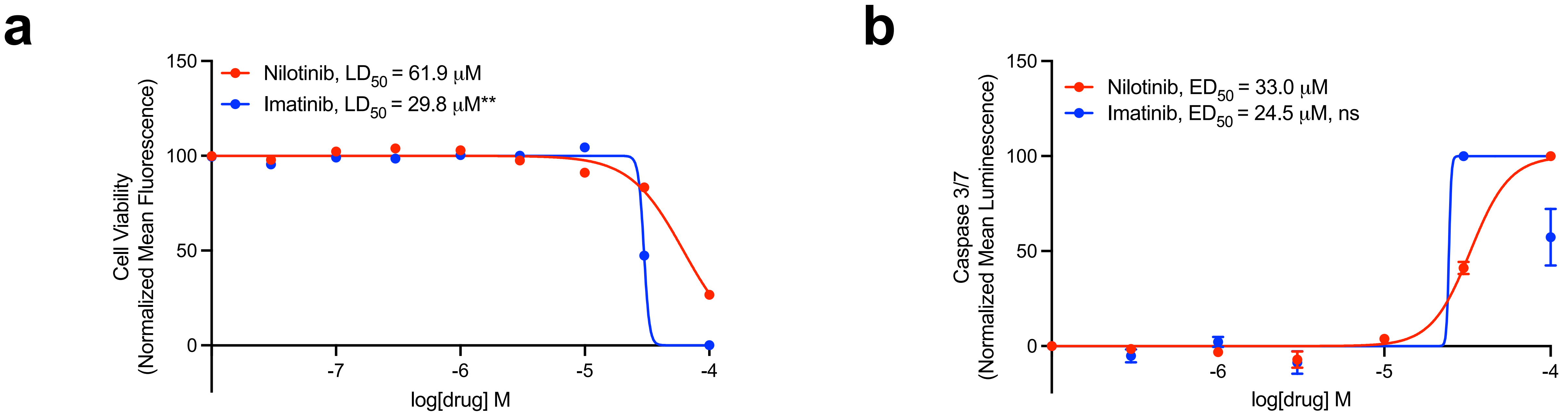

Supplementary Figure 2. Determination of Appropriate *In Vitro* Dosing. a, Resazurin-based kill curve for half-log doses of nilotinib and imatinib (n=5) **b,** Luminescence-based detection of Caspase 3/7 expression following drug exposure (n=3). *n* = biological replicates, unpaired Student's T-test, $^{**}P \leq 0.01$, ns = not significant.

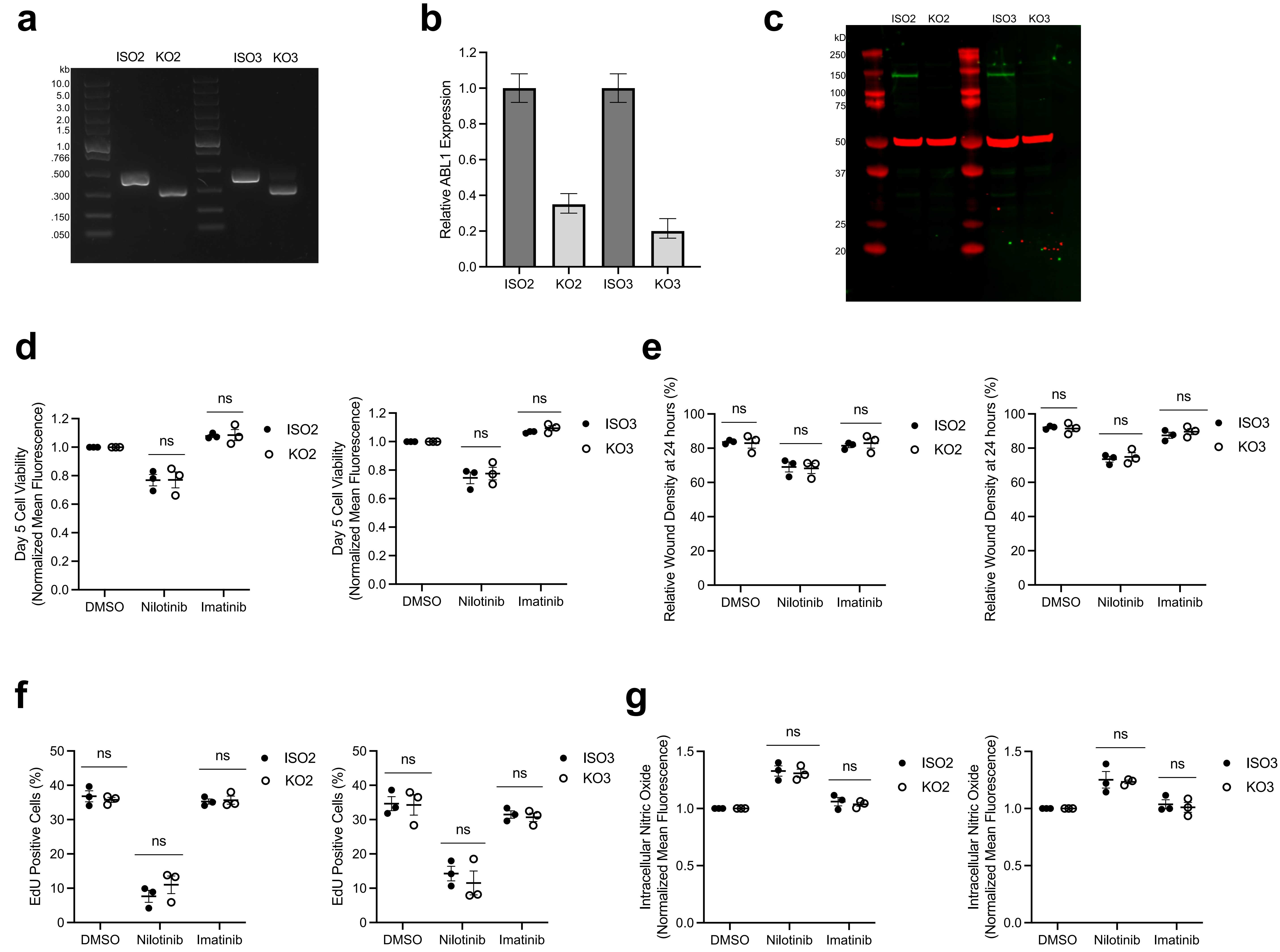

Supplementary Figure 3. Characterization of the Effects of ABL1 Knockout in Two Additional hiPSC Lines. a, DNA Gel Electrophoresis of ISO and ABL1 KO cell lines **b,** RT-PCR of ABL1 expression normalized to ISO cell lines **c,** Western blot for ABL1 in ISO and KO hiPSC-ECs **d**, Resazurin-based assessment of hiPSC-EC Proliferation at Day 5 for ABL1 KO and ISO lines in the presence of 3 μM Drug (n=3) **e**, Comparison of Wound Healing at 24 hours in ABL1 KO and ISO hiPSC-ECs in the presence of 3 μM Drug (n=3) **f**, EdU Nucleoside Analog Incorporation Assay of ABL1 KO and ISO lines in the presence of 3 μM Drug after 24-hour pre-incubation **g**, Flow cytometry analysis of DAF-FM intracellular nitric oxide staining in ABL1 KO and ISO hiPSC-ECs after 72 hours of 3 μM drug exposure (n=3). *n* = biological replicates, unpaired Student's T-test, ns = not significant.

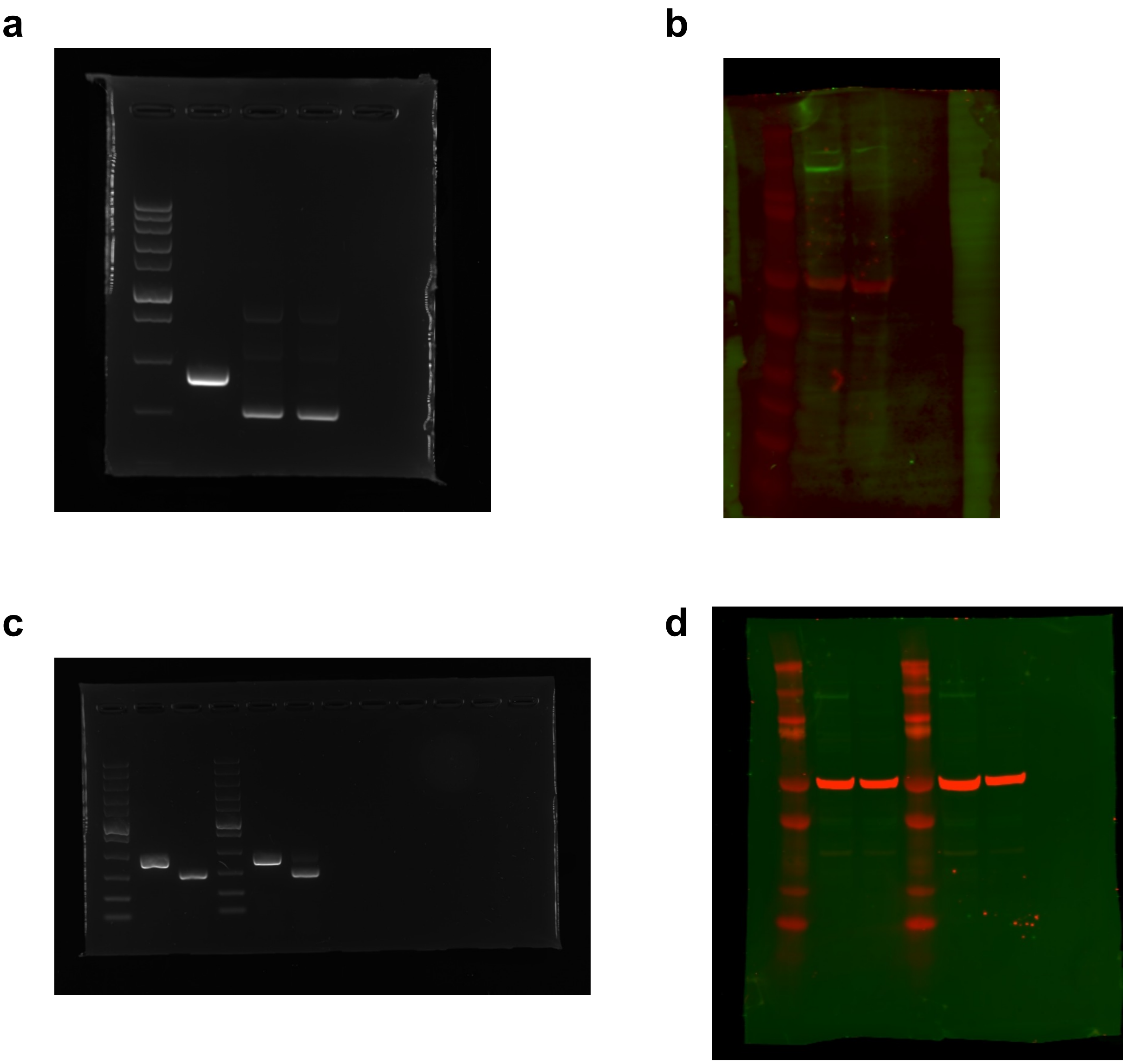

Supplemental Figure 4. Uncropped Blots and Gels with Visible Edges. a, Uncropped DNA Gel Electrophoresis of ISO and ABL1 KO cell lines (corresponding to Figure 4b). Third lane represents a clone that was not ultimately used and hence was cropped out in main figure. **b,** Preliminary Odyssey scan of uncropped Western blot for ABL1 in ISO and KO hiPSC-ECs (corresponding to Figure 4d) **c,** Uncropped DNA Gel Electrophoresis of ISO and ABL1 KO in 2 additional cell lines (corresponding to Supplemental Figure 3a) **d**, Preliminary Odyssey scan of uncropped Western blot of ABL1 KO hiPSC-ECs in 2 additional cell lines (corresponding to Supplemental Figure 3c).

Assay	Target	Manufacturer	Product Number
Flow	CD31-647	BD Biosciences	558094
IF	CD31	BD Biosciences	555444
Flow/IF	SM22/TAGLN	Abcam	ab14106
Flow/IF	Calponin	Sigma	C2687
Flow/IF	α-SMA	eBiosciences	14-9760-82
Flow/IF	MYH11	Abcam	ab82541
Flow/IF	Goat Anti-Mouse IgG1	BD Biosciences	557721
Flow/IF	Goat Anti-Rabbit IgG	Invitrogen	A32740
IF	Goat Anti-Mouse IgG	Invitrogen	A-11005
WB	ABL1	Cell Signaling Technology	2862
WB	β-Tubulin	Sigma	T2200
WB	Goat Anti-Rabbit	LI-COR	926-32211
WB	Goat Anti-Mouse IgG	Invitrogen	35519

Supplementary Table 1. Antibodies

Guide 1	CATCACGCCAGTCAACAGTC
Guide 2	CCATCTCGCTGAGATACGAA
Fwd Primer	AAGCTGGTTTCCAAAGCTGA
Rev Primer	CTTCTTGGTTGAGCTTTCTTGC

Supplementary Table 2. Guides and Primers Used to Generate ABL1 KO Lines
